# *In vitro* and *in vivo* efficacy of vancomycin against *Elizabethkingia* species and the impact of increased vancomycin MICs

**DOI:** 10.1128/spectrum.02371-25

**Published:** 2025-10-01

**Authors:** Tzu-Wen Huang, Teng-Kuang Yeh, Shu-Yuan Hsu, Mei-Chen Tan, Wei-Cheng Huang, Shu-Chen Kuo

**Affiliations:** 1 Department of Microbiology and Immunology, School of Medicine, College of Medicine, Taipei Medical University 243733, Taipei, Taiwan; 2 Graduate Institute of Medical Sciences, College of Medicine, Taipei Medical University 662539, Taipei, Taiwan; 3 Institute of Biotechnology and Pharmaceutical Research, National Health Research Institutes, Zhunan, Taiwan; 4 National Institute of Infectious Diseases and Vaccinology, National Health Research Institutes, Zhunan, Taiwan; 5 Department of Biological Science & Technology, National Yang Ming Chiao Tung University 720928, Hsinchu, Taiwan; University of Guelph College of Biological Science, Guelph, Ontario, Canada

**Keywords:** vancomycin, *in vivo*, efficacy, *Elizabethkingia*

## Abstract

This study aimed to evaluate the concordance of vancomycin susceptibility testing methods, its *in vivo* and *in vitro* efficacy, and the mechanisms underlying elevated MICs in *Elizabethkingia* spp. Vancomycin susceptibilities of 18 *E. anophelis* isolates were determined using multiple assays. The efficacy of vancomycin against five clinical isolates and one laboratory-induced mutant with an elevated vancomycin MIC was evaluated using time-kill assays and *Galleria mellonella* and murine models. Vancomycin MICs (16–32 mg/L) determined by broth microdilution were consistent with agar dilution, Etest, and MBC assay results. All isolates had zone diameters < 17 mm and were, thus, categorized as non-susceptible according to the CLSI criteria for *Enterococcus* spp. Time-kill assays of five clinical isolates demonstrated that vancomycin at a clinically relevant concentration (4 mg/L) exhibited poor bactericidal activity similar to that of teicoplanin. Vancomycin improved *Galleria mellonella* survival in a dose-dependent manner, whereas teicoplanin, dalbavancin, oritavancin, and daptomycin were ineffective. Murine models revealed that vancomycin at a human-equivalent dose (25 mg/kg twice daily) prolonged survival in most infections and modestly reduced bacterial load, while teicoplanin remained ineffective. Vancomycin efficacy was significantly reduced in *G. mellonella* and mice infected with a mutant strain exhibiting an elevated MIC (128 mg/L), which was attributable to spontaneous mutations in *pbp*4. In conclusion, *E. anophelis* were consistently non-susceptible to vancomycin as determined by multiple *in vitro* assays. However, vancomycin demonstrated unique *in vivo* activity among glycopeptides although this effect was abrogated by spontaneous mutations leading to elevated MICs.

**IMPORTANCE:**

*Elizabethkingia anophelis* is a multidrug-resistant pathogen associated with limited treatment options and high mortality. Most commonly considered agents, including fluoroquinolones, piperacillin/tazobactam, and trimethoprim/sulfamethoxazole, are increasingly compromised by resistance, toxicity, or inconsistent efficacy. Although vancomycin is not routinely used for Gram-negative infections due to limited outer membrane permeability, case reports have suggested potential benefit in *Elizabethkingia* infections under critical conditions. In this study, we show that *E. anophelis* isolates are uniformly non-susceptible to vancomycin *in vitro* and exhibit minimal bactericidal activity. However, vancomycin conferred a modest but statistically significant survival benefit in two independent animal models. Importantly, this effect was lost in strains with vancomycin-induced MIC elevation, and genome analysis identified *pbp4* mutations as a potential underlying mechanism. These findings suggest vancomycin may offer therapeutic benefit when no preferred options are available. They support cautious use in selected cases and highlight the need for continued monitoring of susceptibility and resistance development.

## INTRODUCTION

*Elizabethkingia* spp. are ubiquitous non-fermentative aerobic Gram-negative bacilli that cause increasingly frequent nosocomial outbreaks of opportunistic infections especially in the United States and Asia (1). These pathogens are intrinsically resistant to both conventional and novel antibiotics, including β-lactams and carbapenems. Only a few agents, i.e., fluoroquinolones, minocycline, trimethoprim/sulfamethoxazole (TMP/SMX), and piperacillin/tazobactam, have shown retained *in vitro* activity. However, each of these agents has major limitations in clinical use.

Fluoroquinolones were initially considered first-line agents with high susceptibility, but resistance has rapidly emerged due to mutations in *gyrA* and *parC* (1). Minocycline, although supported by our previous *in vivo* data (2), is bacteriostatic and often considered insufficient as monotherapy in critically ill patients. TMP/SMX is associated with considerable toxicity—including bone marrow suppression and renal impairment, limiting its applicability in fragile populations. In addition, susceptibility to TMP/SMX is inconsistent across studies (3). Our prior work has demonstrated that piperacillin/tazobactam exhibits intrinsic resistance**,** lacks bactericidal activity *in vivo*, and is difficult to interpret due to MIC variability (4).

Because the treatments mentioned above are either less effective or limited by toxicity and resistance, vancomycin has occasionally been used as a last-resort agent in *Elizabethkingia* infections, especially in critically ill patients with limited options. Vancomycin inhibits cell wall synthesis by targeting the D-Ala-D-Ala peptide terminus of peptidoglycan and is ineffective against Gram-negative bacilli due to its poor penetration of the cell wall (5). However, vancomycin mono- and combination therapies of pediatric *Elizabethkingia* infections have yielded some success (6). Intriguingly, these clinical observations contrast with consistently high vancomycin MICs. In addition, previous *in vitro* studies of vancomycin susceptibilities of *Elizabethkingia* spp. reported discrepant results between broth microdilution and disk diffusion assays (6).

To address these uncertainties, this study aimed to evaluate vancomycin susceptibility using multiple *in vitro* assays and to assess its *in vivo* activity. We also investigated the genetic basis of elevated vancomycin MICs through induced mutation and genome analysis. Our goal was not to position vancomycin as a first-line therapy but to clarify its potential utility in specific clinical scenarios where standard treatments are limited or ineffective.

## MATERIALS AND METHODS

### Selection of experimental isolates

Eighteen *E. anophelis* clinical isolates from the nationwide surveillance program (Taiwan Surveillance of Antimicrobial Resistance) were well characterized in our previous study (7) and were selected for susceptibility testing and minimum bactericidal concentration (MBC) assays. Among them, five isolates with different clonality (underlined strains in Fig. S1) were selected for the time-kill assays and *in vivo* experiments.

### Susceptibility testing, MBC test, and time-kill assay

Broth microdilution, disc diffusion, Etest, and agar dilution assays were conducted following CLSI guidelines to determine vancomycin MICs (8). Due to the lack of breakpoints for *Elizabethkingia* spp., criteria for *Enterococcus* spp. were adopted (susceptibility, MIC ≤ 4 mg/L and zone diameter ≥ 17 mm) (9). Criteria for *Staphylococcus* spp. were not adopted due to the lack of a zone diameter breakpoint. MBCs were determined following CLSI guidelines (8).

Time-kill assays were performed as previously described with modifications (10). Each 250 mL flask containing 30 mL MH broth (Becton Dickinson, Franklin Lakes, NJ, USA) was inoculated to achieve an initial bacterial density of ~5 × 10⁵ CFU/mL. Cultures were incubated at 37°C with shaking at 200 rpm. Vancomycin or teicoplanin (Sigma-Aldrich, Saint Louis, MO, USA) was added at final concentrations of 4 or 16 mg/L. At designated time points, 100 µL of samples was removed, serially diluted in sterile saline, and plated on MH agar for CFU enumeration after overnight incubation at 37°C. No washing steps were performed prior to plating; given the culture volume and dilution scheme, antibiotic carryover was expected to be negligible. All experiments were performed in triplicate.

### *In vivo* efficacy of glycopeptides and daptomycin

*In vivo* efficacy was assessed by wax moth (*Galleria mellonella*), murine pneumonia, and murine thigh infection models following the protocols of our previous study (2). The Animal Ethics Committee of Taiwan’s National Health Research Institutes approved the murine pneumonia and thigh infection models.

*G. mellonella* was reared at the National Health Research Institutes of Taiwan. Caterpillars in the final instar larval stage (weight ca. 250 mg) were used. For each infecting strain, dose-ranging experiments were performed to determine the lowest inoculum that resulted in >80% mortality within 72 h; this inoculum was used for subsequent efficacy testing. A 10 µL aliquot of *E. anophelis* at the predetermined lethal dose was injected into the hemocoel of each caterpillar via the last left proleg. Antibiotics were administered once into another proleg at the indicated time points; vancomycin (5, 10, or 20 mg/kg, 2 h post-inoculation); other glycopeptides (teicoplanin, 10 mg/kg, 30 min post-inoculation; dalbavancin, 8.75 mg/kg, 2 h post-inoculation; oritavancin, 15 mg/kg, 1 h post-inoculation) (11–13); or daptomycin (10 mg/kg, 2 h post-inoculation) (13). Each treatment group contained 6–8 caterpillars. A control group injected with sterile PBS was included to monitor for trauma-related mortality. Caterpillars were incubated at 37°C, and survival was recorded twice daily for 72 h.

For the pneumonia model, a bacterial suspension was mixed with 5% mucin from porcine stomach (type 3; Sigma-Aldrich, Saint Louis, USA). Seven-week-old C57BL/6J mice were anesthetized with isoflurane for 10 min. For each strain, the inoculum corresponded to the lowest dose that caused >80% mortality in preliminary testing. This inoculum (10⁶–10⁸ cfu, depending on strain) was delivered intratracheally to induce pneumonia. Two hours thereafter, mice received either high or human-equivalent doses of subcutaneous vancomycin (100 mg/kg [14, 15] or 25 mg/kg, respectively [16]) or intraperitoneal teicoplanin (50 mg/kg) (17) twice daily for 3 days. Each group contained eight mice, and survival was monitored for 3 days.

For the thigh infection model, seven-week-old C57BL/6J mice were rendered neutropenic by intraperitoneal injections of cyclophosphamide (Cytoxan; Bristol-Myers Squibb, Princeton, NJ, USA) 4 days (150 mg/kg) and 1 day (100 mg/kg) before bacterial inoculation. A 30 µL aliquot containing the *E. anophelis* (10^7^ cfu) was injected into each of the two rear thighs. Vancomycin (25 mg/kg or 100 mg/kg, subcutaneous) or teicoplanin (50 mg/kg, intraperitoneal) was administered 2 h and 14 h after inoculation. Mice were sacrificed 24 h post-inoculation, and homogenized thigh tissue was plated in serial 10-fold dilutions on brain-heart infusion agar to determine bacterial counts. Each mouse, not each thigh, was considered an individual data point.

### Determination of serum drug levels in mice

Fifty microliters of aliquots of murine serum was mixed with 100 µL acetonitrile. The mixture was vortexed and then centrifuged at 15,000 × *g* for 20 min in a Beckman Coulter Microfuge 22R Centrifuge at room temperature. The supernatant was transferred to a clean tube, and then 15 µL of the supernatant was injected onto LC-MS/MS. The chromatographic system consisted of an Agilent 1200 series LC system and an Agilent ZORBAX Eclipse XDB-C_8_ column (5 µm, 3.0 × 150 mm) interfaced to an MDS Sciex API3000 tandem mass spectrometer. The MS/MS ion transitions monitored were *m*/*z* 725/144 for vancomycin. A gradient HPLC method was employed for separation. Mobile phase A consisted of 10 mM ammonium acetate aqueous solution containing 0.1% formic acid and mobile phase B consisted of acetonitrile. The gradient profile was as follows (min/%B): 0.0–0.9/5, 1.0–2.0/50, 2.1–3.7/95, 3.8–5.0/5. The flow rate was set at 1.5 mL/min.

### Induction of vancomycin MIC elevation and validation of resistance mechanisms

*E. anophelis* and non-anophelis species with baseline vancomycin MICs of 16–32 mg/L (Table 2) were well characterized in our previous study (7) and were included in the induction study. The induction of mutants with elevated vancomycin MICs was modified from a previous study (18). Briefly, 10^9^ cfu of wild-type *E. anophelis* (2008S01-229) was plated on brain heart infusion agar (Becton Dickinson, Franklin Lakes, NJ, USA) with twofold multiples of vancomycin (32 –256 mg/L). Isolates from viable colonies were subjected to broth microdilution. Genomic comparison between the parental strain (2008S01-229) and elevated-MIC mutants was performed to identify the mutated gene conferring increased vancomycin MIC. To determine whether similar genetic changes occurred in other *Elizabethkingia* species, non-anophelis isolates were subjected to the same induction protocol. The corresponding target genes were then analyzed by Sanger sequencing to assess the presence of analogous mutations. The target gene related to elevated vancomycin MICs was also validated via scarless gene editing.

### Scarless gene editing

The suicide plasmid pUT-ermF, which contains two key selection markers: the *erm*F gene for erythromycin resistance and the *sac*B gene for sucrose sensitivity-based counterselection, was used as previously described (19). DNA fragments corresponding to the upstream and downstream flanking regions of the target gene were cloned into pUT-ermF. The recombinant plasmids were first introduced into *E. coli* S17–λpir and subsequently transferred into *Elizabethkingia* spp. via conjugation. The first recombination events were selected on LB agar containing 250 µg/mL erythromycin, and the second recombination (plasmid resolution) was selected by plating on LB agar with 10% sucrose (without NaCl). Colonies were screened and confirmed by PCR sequencing. The primers used for gene editing are listed in Table S1. Genome sequencing was carried out to confirm no other inadvertent changes.

### Genome sequencing and comparison

Whole-genome sequencing was conducted using Oxford Nanopore Technologies (ONT) long reads. The DNA library for ONT MinION sequencing was constructed using the ONT rapid barcoding kit (SQK-RBK114-96) and then loaded into a MinION SpotON flowcell R10.4.1 (FLO-MIN114). The sequencing script was executed on MinKnow v25.03.7, and live base calling was performed using Dorado v7.8.3. super accuracy model for sequencing read collection. Genomes were *de novo* assembled using flye v2.9.4-b1799 and polished with medaka v2.0.1. Sequences of the parental strain and mutants with elevated vancomycin MIC or gene-edited mutants were compared by manual basic local alignment search tool (BLAST) analysis to identify mutations.

### Data analysis

Kaplan–Meier survival analysis (log-rank test) was used to assess differences in survival. Student’s *t*-test was used to compare bacterial loads. GraphPad Prism (version 6; GraphPad Software) was used for statistical analyses.

## RESULTS

### Susceptibility and MBCs

MICs determined by broth microdilution were concordant with those obtained by agar and Etest dilution assays, which were within 16–32 mg/L (Table 1). All isolates had zone diameters < 17 mm. All susceptibility tests showed that *Elizabethkingia* spp. were resistant to vancomycin based on criteria for *Enterococcus* spp. MBCs were within a twofold range of MICs. The MICs of teicoplanin were 4- to 8-fold higher than those of vancomycin.

**TABLE 1:** Results of different susceptibility tests and minimum bactericidal concentrations of 18 *Elizabethkingia anophelis* isolates^*b*^

	Vancomycin (mg/L or mm)					Teicoplanin (mg/L)
	BMD	MBC	Etest	AD	DD^*a*^	BMD
2008S01-229	32	32	16	16	15	128
2008N05-106	32	16	24	16	15	128
2014C06-157	32	16	16	16	15	128
2016N17-576	32	32	16	16	15	128
2018C04-213	16	16	16	16	15	128
2018C04-224	16	16	16	16	15	128
2018S09-023	16	16	16	16	15	64
2018E03-151	16	16	16	16	14	128
2018C07-210	16	16	16	16	14	256
2018C07-234	16	16	16	16	15	256
2018N16-057	16	16	16	16	15	128
2018N17-036	32	32	16	16	15	128
2018N17-073	16	16	16	16	14	128
2018N17-153	16	16	16	16	14	128
2018C08-127	16	16	16	16	14	128
2018S11-172	32	32	32	32	13	64
2018N21-226	16	16	24	16	13	64
2018E04-135	16	16	16	16	14	64

^*a*^ mm.

^*b*^ BMD, broth microdilution; MBC, minimum bactericidal concentration; AD, agar dilution; DD, disc diffusion.

### Time-kill assay

Vancomycin at a clinically relevant concentration (4 mg/L) did not inhibit the growth of any of the five tested clinical isolates (Fig. S2a). Growth was inhibited at a higher level (16 mg/L) at 6 h but increased thereafter. A similar killing pattern was observed in isolates treated with teicoplanin at the same concentration (Fig. S2b).

### *In vivo* efficacy of vancomycin monotherapy

**Fig 1:**
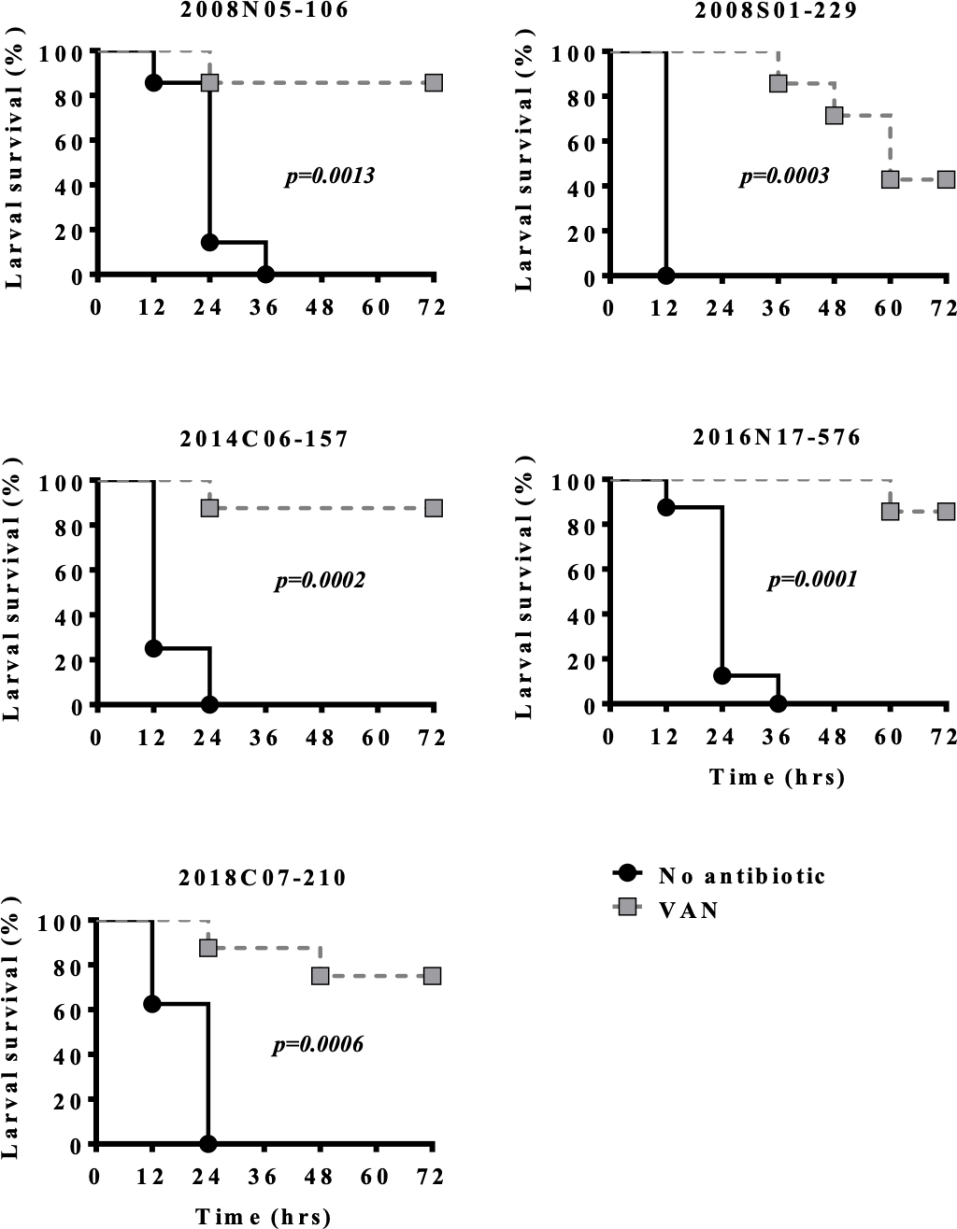
Efficacy of vancomycin (VAN) against *Elizabethkingia anophelis* in *Galleria mellonella* assays. A 10 µL aliquot of *E. anophelis* was injected via the last left proleg. Two hours after inoculation, each caterpillar was injected with 10 µL PBS with or without vancomycin (10 mg/kg) via the last right proleg, and survival was observed for 3 days at 37°C. The survival rate of the trauma control group of uninfected caterpillars injected with PBS was 100%. Each treatment group contained 6–8 caterpillars.

Vancomycin improved the survival of wax moths infected by all five *E. anophelis* isolates (Fig. 1). A dose-dependent effect was also observed in two clinical isolates (Fig. S3). In contrast, other glycopeptides, including teicoplanin, dalbavancin, and oritavancin, as well as daptomycin, conferred no survival benefit (Fig. S4).

**Fig 2:**
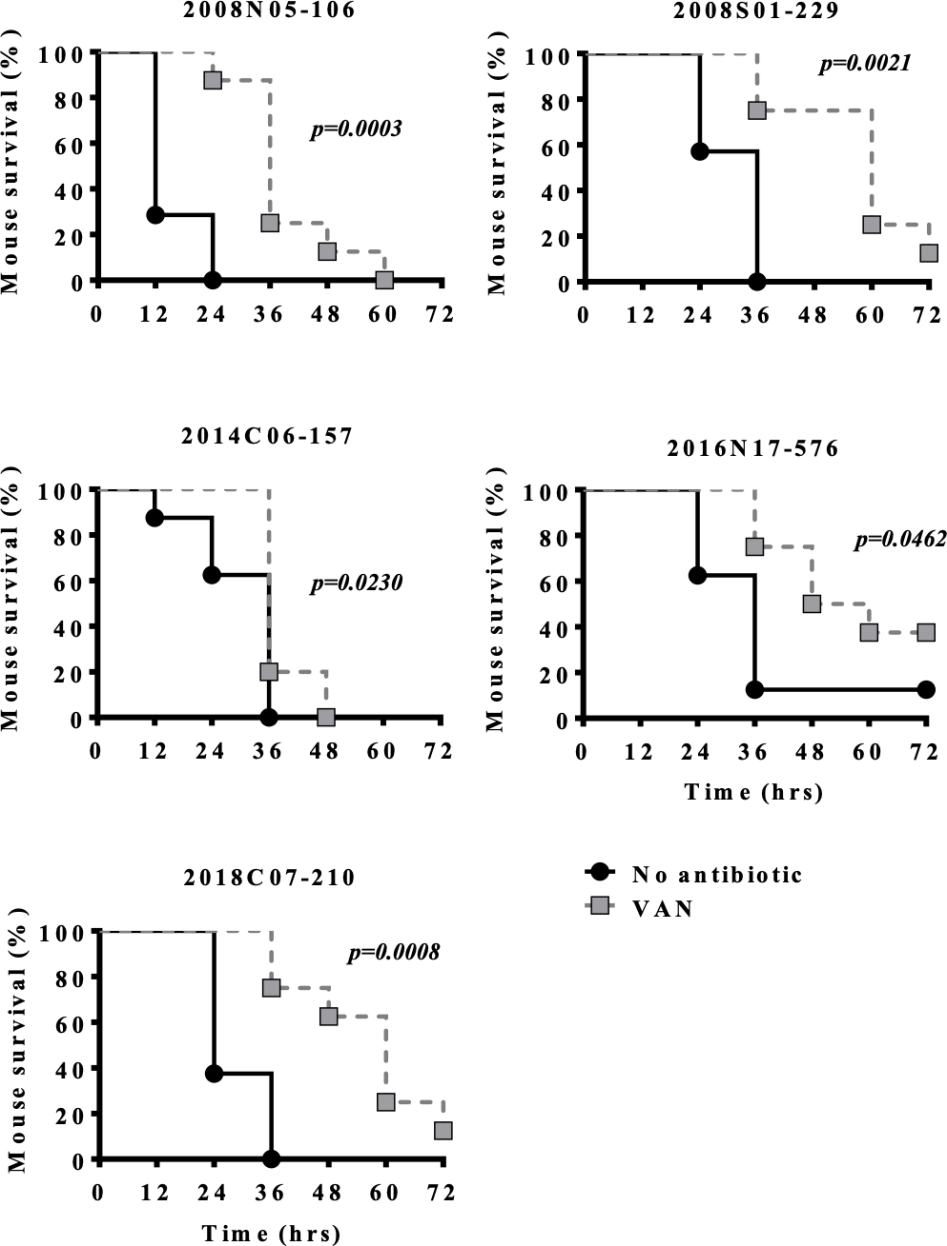
Efficacy of vancomycin (25 mg/kg twice daily) (VAN) against *Elizabethkingia anophelis* in a murine pneumonia model. Seven-week-old C57BL/6J mice underwent intratracheal inoculation with *E. anophelis*. Two hours later, the mice were injected subcutaneously with PBS or vancomycin for 3 days. Each treatment group contained 8 mice.

Vancomycin (25 mg/kg) significantly improved the survival or delayed death of mice infected by all clinical strains except 2014C06-157 (Fig. 2) and modestly reduced the bacterial loads of all clinical strains (Fig. S5). To determine whether *in vivo* vancomycin exposures may lead to elevated vancomycin MICs, isolates from the thigh infection model were plated on agar containing vancomycin (32 mg/L), and no viable colony was observed. High-dose (100 mg/kg) vancomycin treatment also conferred a survival benefit (Fig. S6) and a greater reduction of bacterial load (Fig. S7). The serum concentration-time profile after a single dose of vancomycin (25 mg/kg) resembled that of humans in contrast to that of the 100 mg/kg dose (Fig. S8).

The validity of our murine pneumonia and thigh infection models was evaluated by treating *E. anophelis* infections with teicoplanin and *A. baumannii* infections with vancomycin. Teicoplanin had little effect on survival or reduction of *E. anophelis* bacterial loads (Fig. S9). High-dose vancomycin (100 mg/kg) did not improve the survival of *A. baumannii-*infected mice (Fig. S10).

### Mechanisms of increased vancomycin MICs

Induction experiments of *E. anophelis* successfully yielded several mutants (Table 2). Genome comparison with parental strains showed that mutations in *pbp*4 and/or *exb*D may increase vancomycin MICs in *E. anophelis*. Mutations of *pbp*4 were identified in other *Elizabethkingia* spp. with elevated vancomycin MICs by Sanger sequencing (Table 2). Because most alterations (i.e., frameshift or nonsense mutations) of *pbp4* cause loss of function, scarless deletion of *pbp4* in wild-type *E. anophelis* (2008N05-106) was conducted and resulted in an MIC increase from 32 to >512 mg/L. The *exb*D mutant (Thr28Pro) was associated with an increased MIC (Table 2). However, replacement of *exb*D with the same mutation in 2008N05-106 did not affect the vancomycin MIC.

**TABLE 2:** Laboratory-induced mutants of *Elizabethkingia* spp. with elevated vancomycin MICs and mechanisms^*a*^

Parent strain (MIC, mg/L)	Gene	Type of mutation		MIC (mg/L)	No. of isolates
		Nucleotide change	Amino acid change		
*E. anophelis* (32)	*pbp*4	C64T	Stop	128	1
		G517A	Gly173Arg	256	1
		827DelA	Frameshift	128	1
		736Del8bp	Frameshift	512	1
		692InsC	Frameshift	512	1
		406Del474bp	Frameshift	128, 512	2
	*exb*D	A82C	Thr28Pro	128–512	4
*E. meningoseptica* (16)	*pbp*4	G437A	Gly146Glu	64	1
		827DelA	Frameshift	64, 128	2
		C777G	Stop	128	1
		C664T	Stop	256	1
		C434T	Ser145Phe	256	1
		G397T	Stop	256	1
*E. ursingii* (32)	*pbp*4	G725T	Gly242Val	256	1
		G725A	Gly242Asp	256	1
*E. bruuniana* (16)	*pbp*4	G436T	Gly146Cys	256	2
*E. miricola* (32)	*pbp*4	66Del72bp	Frameshift	512	3

^*a*^ 10^9^ cfu of wild-type *Elizabethkingia* spp. were plated on brain heart infusion agar with two-fold multiples of vancomycin (32 to 256 mg/L). Isolates from viable colonies were subjected to broth microdilution. Whole-genome sequencing was performed on *E. anophelis *mutants displaying a ≥4-fold increase in vancomycin MIC and their parental strains, while Sanger sequencing was conducted for corresponding mutants and parental strains of other *Elizabethkingia* species.

### Impact of elevated vancomycin MICs

**Fig 3:**
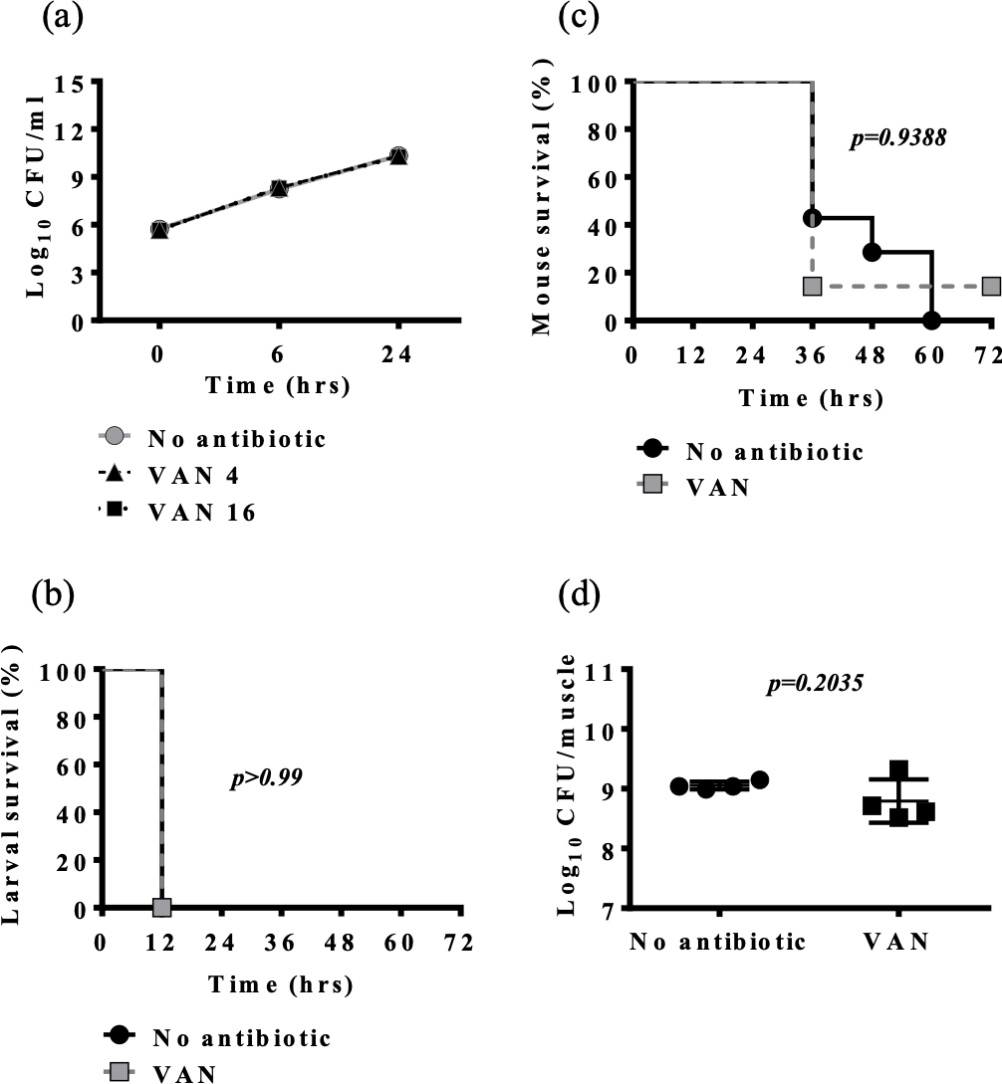
*In vitro* and *in vivo* efficacy of vancomycin (VAN) against *Elizabethkingia anophelis* with elevated vancomycin MIC (2008S01-229-VAN-R, MIC 128 mg/L). (**a**) Time-kill assay results obtained with 4 mg/L (VAN 4) and 16 mg/L (VAN 16). (**b**) *G. mellonella* assay results obtained with 10 mg/kg VAN. (**c**) Murine pneumonia results obtained with 100 mg/kg VAN twice daily. (**d**) Thigh infection model results obtained with 100 mg/kg VAN twice daily. Time kill assays were performed in triplicate. Due to the very low inter-replicate variability, error bars are not shown.

*In vitro* and *in vivo* efficacies of vancomycin were tested in the 2008S01-229 mutant (2008S01-229-VAN-R, vancomycin MIC of >512 mg/L). Time-kill assays showed no effect of vancomycin at 4 or 16 mg/L (Fig. 3). Vancomycin-mediated improvement of murine and larval survival and reduction of bacterial load was hampered in animals infected by 2008S01-229-VAN-R (Fig. 3) compared to 2008S01-229 (vancomycin MIC 32 mg/L, Fig. 1 and 2).

## DISCUSSION

Most *in vitro* active agents such as minocycline, levofloxacin, and TMP/SMX exhibited lower MICs against *E. anophelis* (Table S2), but each has notable clinical limitations as previously discussed. These constraints highlight the need to consider alternative or adjunctive therapies such as vancomycin, particularly in difficult-to-treat cases. This study demonstrated concordant vancomycin susceptibility results among different *in vitro* assays, absent *in vitro* bactericidal activity at a clinically relevant concentration, and significant *in vivo* efficacy of vancomycin against *Elizabethkingia* spp. The elevation of MICs after *in vitro* exposure to vancomycin was associated with alterations of *pbp4* and reduced *in vivo* efficacy.

MIC results obtained by different methods, i.e., broth microdilution, agar dilution, and Etest assays, were highly congruent and were in accordance with MBC values. Using the current breakpoint of *Enterococcus* spp. proposed by CLSI (9), our MIC testing and disc diffusion assays concurrently showed non-susceptibility of all *E. anophelis* isolates. The discordance of the susceptibility results between broth microdilution and diffusion methods reported in the previous literature may be due to the adoption of the breakpoint of a diffusion method for *Staphylococcus* spp. that was subsequently abolished (6); using the old breakpoint (zone diameter ≥12 mm) (6), the results from disc diffusion would identify all of our 18 isolates as being susceptible, in conflict with MIC results. The vancomycin non-susceptibility of *Elizabethkingia* spp. aligned with the results from our time kill assay. Bactericidal activity was only prominent at high (16 mg/L) but not clinically relevant (4 mg/L; breakpoint of *Enterococcus* spp.) concentrations.

Our time-kill assay showed vancomycin and teicoplanin had similar *in vitro* efficacy; however, vancomycin demonstrated its unique *in vivo* efficacy compared to other glycopeptides in terms of improving survival of *G. mellonella*. In addition, both high and low dose vancomycin improved the survival of most *E. anophelis-*infected mice; in contrast, teicoplanin was ineffective in mice. The unique *in vivo* efficacy of vancomycin cannot be simply explained by its lower MICs (32 mg/L) compared to teicoplanin (64–128 mg/L) because the teicoplanin concentration in murine serum was much higher than that of vancomycin (Fig. S8). The mechanisms underlying the discrepancy of *in vivo* efficacy between vancomycin and other glycopeptides merit further investigation.

The efficacy of vancomycin against *Elizabethkingia* spp. has been observed in zebra fish and an *ex vivo* precision-cut lung slice model (20, 21). Our study provides further evidence of its effect in wax moth and murine models. The discrepancy of vancomycin doses used in previous murine studies (15, 16) prompted us to question which doses best replicated human vancomycin pharmacokinetics. Compared to the results of a previous study that assessed vancomycin pharmacokinetics in 10 adult cystic fibrosis patients given a single dose of vancomycin (15 mg/kg) for acute bronchopulmonary exacerbation (22), the murine pharmacokinetics of a 25 mg/kg vancomycin dose observed in this study may bear more resemblance to human pharmacokinetics than those of a 100 mg/kg dose (Fig. S8). Vancomycin at 25 mg/kg improved survival or delayed death in infections due to 4 of the 5 tested *E. anophelis* strains. Good efficacy in multiple animal experiments and our pharmacokinetic results aligned with the favorable clinical effectiveness observed in several previous reports (6). However, vancomycin was ineffective *in vivo* against one isolate with MICs that were similar to those of other isolates. The identification of other bacterial factors associated with treatment efficacy is of clinical importance but beyond the scope of our study.

Vancomycin exhibits low potency against Gram-negative pathogens due to impermeability of the outer membrane to large molecules (5). Its lack of clinical utility in Gram-negative infections has limited investigations of the mechanisms underlying its high vancomycin MIC. Our study revealed that alteration of *pbp*4 elevated vancomycin MICs in *Elizabethkingia* spp. The loss of PBP activity has been associated with increased levels of monomeric muropeptides carrying the target of vancomycin, carboxyl-terminal D-alanyl–D-alanine residues, which allows the capture of vancomycin and protects *Staphylococcus aureus* (23, 24). Fortunately, our study did not detect isolates with elevated MICs after vancomycin treatment at the human equivalent dose in our thigh infection model. This may imply that spontaneous mutation in *Elizabethkingia* spp. may not be expected in the clinical setting; however, the risk exists.

In conclusion, this study found good agreement among different susceptibility testing methods for vancomycin in *Elizabethkingia* spp. MIC testing and disc diffusion results were concordant when using the *Enterococcus* spp. breakpoint proposed by CLSI. Vancomycin demonstrated unique *in vivo* activity against *Elizabethkingia* spp. in contrast to its poor *in vitro* activity. This may explain its previously observed clinical effectiveness. The elevation of MICs following vancomycin exposure was associated with *pbp*4 alterations. While vancomycin is not recommended for routine use, these findings suggest it may warrant cautious consideration in cases where no preferred treatment options are available, with close monitoring for resistance development.

## Data Availability

The genome sequences of isolates 2008S01-229 (CP077752), 2008S01-229-VAN-R (CP198982), 2008N05-106 (CP198981), 2008N05-106_pbp4 (CP198980), and 2008N05-106_exbD (CP198979) have been deposited in GenBank.

## SUPPLEMENTAL MATERIAL

Supplemental material spectrum.02371-25-s0001.docx Tables S1 and S2; Fig. S1 to S10.

ASM does not own the copyrights to Supplemental Material that may be linked to, or accessed through, an article. The authors have granted ASM a non-exclusive, world-wide license to publish the Supplemental Material files. Please contact the corresponding author directly for reuse.
