# Supplementary material for: In vitro and in vivo efficacy of vancomycin against Elizabethkingia species and the impact of increased vancomycin MICs
Source: Microbiol Spectr. 2025 Oct 1;13(11):e02371-25. doi: 10.1128/spectrum.02371-25 (PMC12584665; doi:10.1128/spectrum.02371-25)

**Table S1 Primers used in this study**

| primer name | primer sequence (5'-3') | purpose |
| --- | --- | --- |
| pUT-PBP4-UP-F | ggcgaattgggccctcaatacaccaagcaatcacc | Amplify truncated *pbp*4 for cloning into the plasmid. |
| PBP4-UP-R | atagtttattcataaatgttttgttataaagaaagag |  |
| PBP4-down-F | taacaaaacatttatgaataaactatatttcatagc |  |
| PBP4-down-pUT-R | cgctcgagcatgcatagccttgtaatgcattgtcg |  |
| PBP4-UF-C | tgcagaaatagagaatcttctc | Used for PCR and sequencing to confirm the truncated *pbp*4 is inserted at the correct locus and the sequence is correct |
| PBP4-DR-C | attatcaggctgtcgtttcg |  |
| pUT-exbD-F | gaattgggccctctagaatggcgagagttaaacc | Amplify *exb*D for cloning into the plasmid |
| exbD-pUT-R | tcgagcatgcatctagattattcgaagttcattgtaacc |  |
| exbD-SDM-F | gtaagggtcgatatgcccccgatggtag | Introduce site-directed mutagenesis of *exb*D on the plasmid |
| exbD-SDM-R | ctaccatcgggggcatatcgacccttac |  |
| exbD-up-CF | gacgaaatcggaatgagcat | Used for PCR and sequencing to confirm the mutated *exb*D is inserted at the correct locus and the sequence is correct |
| exbD-down-CR | tcacaaagatccccaatgtg |  |

**Table S2 Broth microdilution results (mg/L) for minocycline, levofloxacin, and trimethoprim-sulfamethoxazole in *Elizabethkingia anophelis* isolates**

|  | Minocycline | Levofloxacin | Trimethoprim-sulfamethoxazole |
| --- | --- | --- | --- |
| 2008S01-229 | ≤2 | ≤1 | 1 |
| 2008N05-106 | ≤2 | >8 | 1 |
| 2014C06-157 | ≤2 | >8 | 2 |
| 2016N17-576 | ≤2 | >8 | 1 |
| 2018C04-213 | ≤2 | >8 | 1 |
| 2018C04-224 | ≤2 | >8 | 1 |
| 2018S09-023 | ≤2 | ≤1 | 1 |
| 2018E03-151 | ≤2 | 2 | 2 |
| 2018C07-210 | ≤2 | >8 | 1 |
| 2018C07-234 | ≤2 | >8 | 1 |
| 2018N16-057 | ≤2 | ≤1 | ≤0.5 |
| 2018N17-036 | ≤2 | >8 | 2 |
| 2018N17-073 | ≤2 | >8 | 1 |
| 2018N17-153 | ≤2 | >8 | 1 |
| 2018C08-127 | ≤2 | 2 | 1 |
| 2018S11-172 | ≤2 | 2 | 1 |
| 2018N21-226 | ≤2 | ≤1 | 2 |
| 2018E04-135 | ≤2 | ≤1 | 2 |

**Fig. S1 PFGE patterns of *Elizabethkingia anophelis* isolates used in this study.** All strains underwent susceptibility and minimum bactericidal concentration testing. Underlined isolates were subjected to time-kill assays and *in vivo* studies.

**Fig. S2 Time-kill assays of (a) vancomycin at 4 mg/L (VAN 4) and 16 mg/L (VAN 16) and (b) teicoplanin at 4 mg/L (TEC 4) and 16 mg/L (TEC 16) against *Elizabethkingia anophelis* isolates*.*** All experiments were performed in triplicate. Due to the very low inter-replicate variability, error bars are not shown


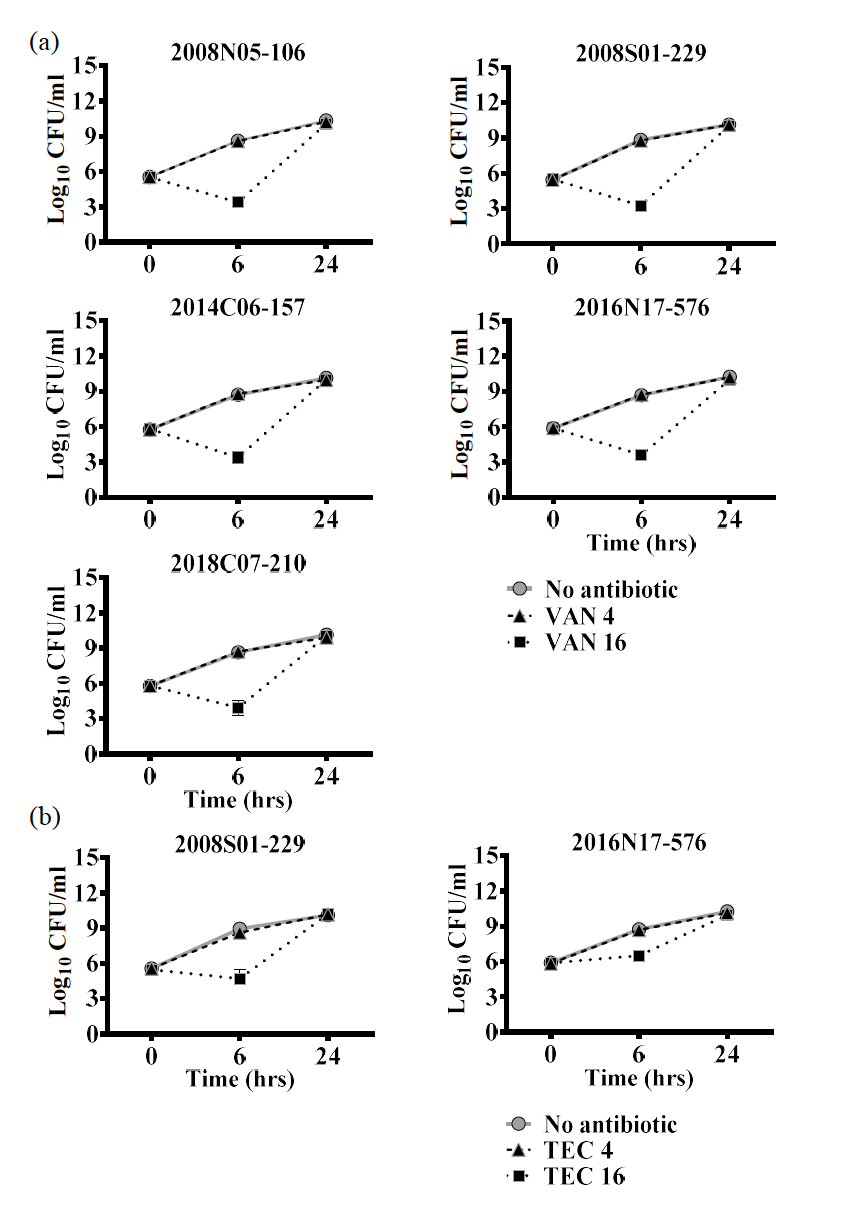


**Fig. S3 Dose-dependent vancomycin (VAN) activity against *Elizabethkingia anophelis* at 5, 10, and 20 mg/kg concentrations in *Galleria mellonella* assays.** A 10 μL aliquot of *E. anophelis* was injected via the last left proleg. Two hours after inoculation, each caterpillar was injected with 10 μL PBS with or without vancomycin via the last right proleg, and survival was observed for 3 days at 37 °C. The survival rate of the control group of traumatized but uninfected caterpillars injected with PBS was 100%. Each treatment group contained 6–8 caterpillars.


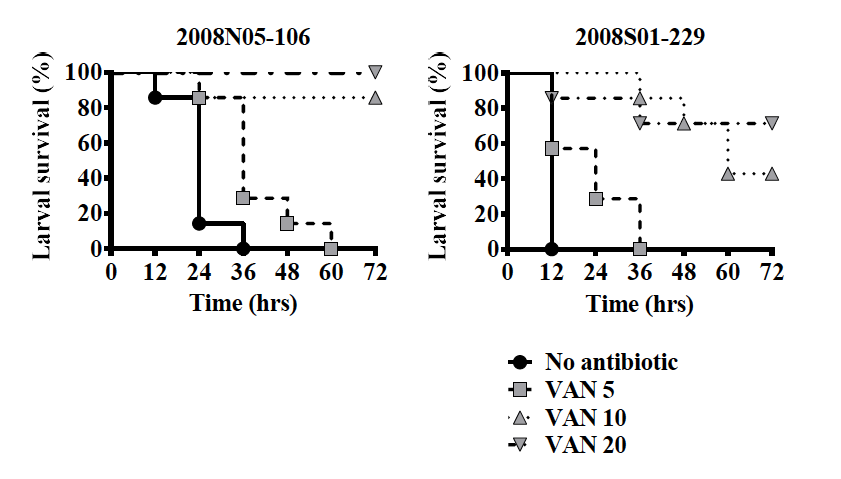


**Fig. S4 Lack of efficacy of other glycopeptides and daptomycin against *Elizabethkingia anophelis* in *Galleria mellonella* assays.** MICs of daptomycin, dalbavancin, oritavancin, and teicoplanin were 128, 64, 64, and 128 mg/L, respectively.

**
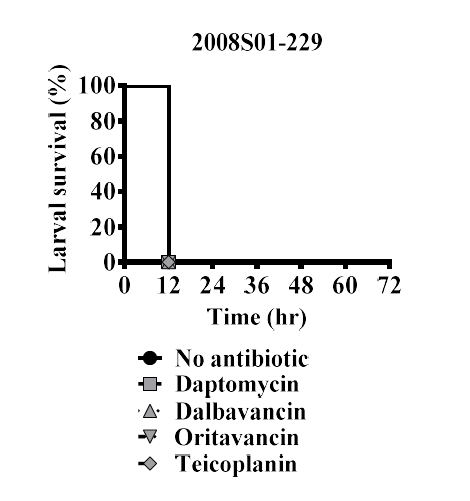
**

**Fig. S5 Bacterial loads of *Elizabethkingia anophelis* in mice given vancomycin (VAN, 25 mg/kg) in a thigh infection model.** Seven-week-old C57BL/6J mice were rendered neutropenic by intraperitoneal injections of cyclophosphamide. Subcutaneous vancomycin (25 mg/kg) was administered 2 h and 14 h after inoculation of *E. anophelis* into each of the two caudal thighs. The mice were sacrificed 24 h after inoculation. Each treatment group contained four mice. Each point represents the bacterial load of each mouse, and the bars indicate means and standard deviations.


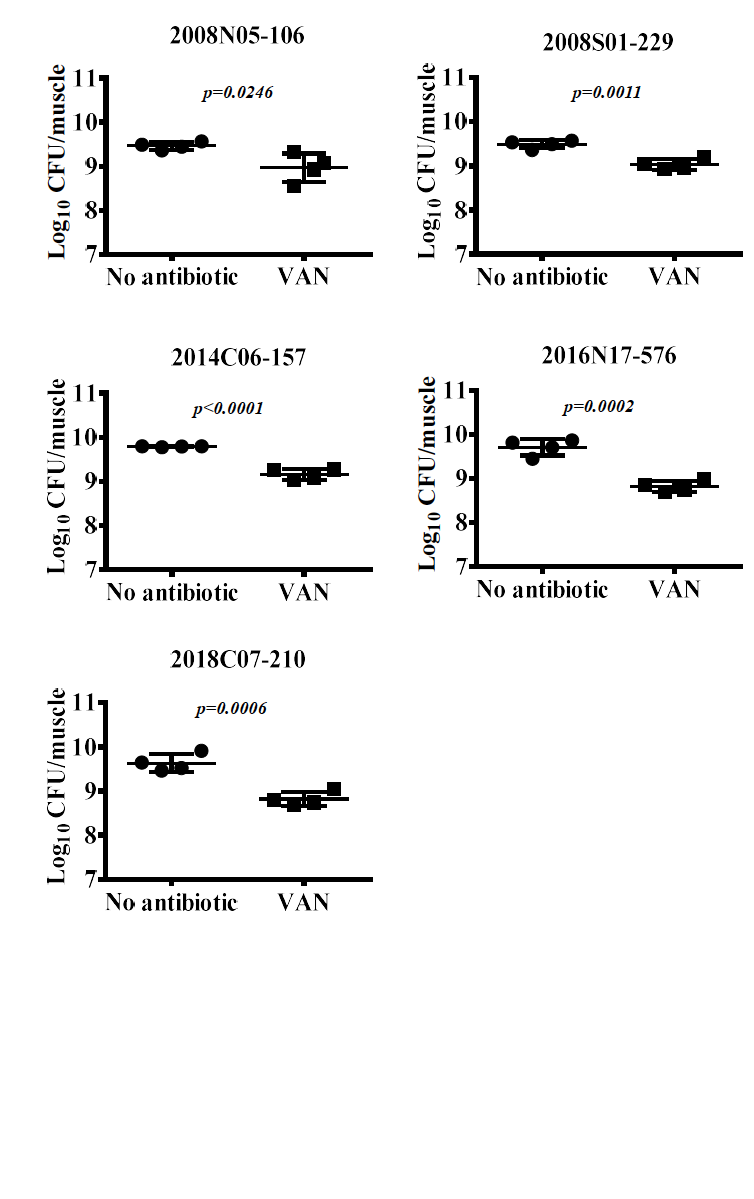


**Fig. S6 Efficacy of high-dose vancomycin (100 mg/kg twice daily, VAN 100) against *Elizabethkingia anophelis* in a murine pneumonia model.** Seven-week-old C57BL/6J mice underwent intratracheal inoculation with *E. anophelis*. Two hours later, the mice were injected subcutaneously with PBS or vancomycin (100 mg/kg) twice daily for 3 days. Each treatment group contained 8 mice.

**
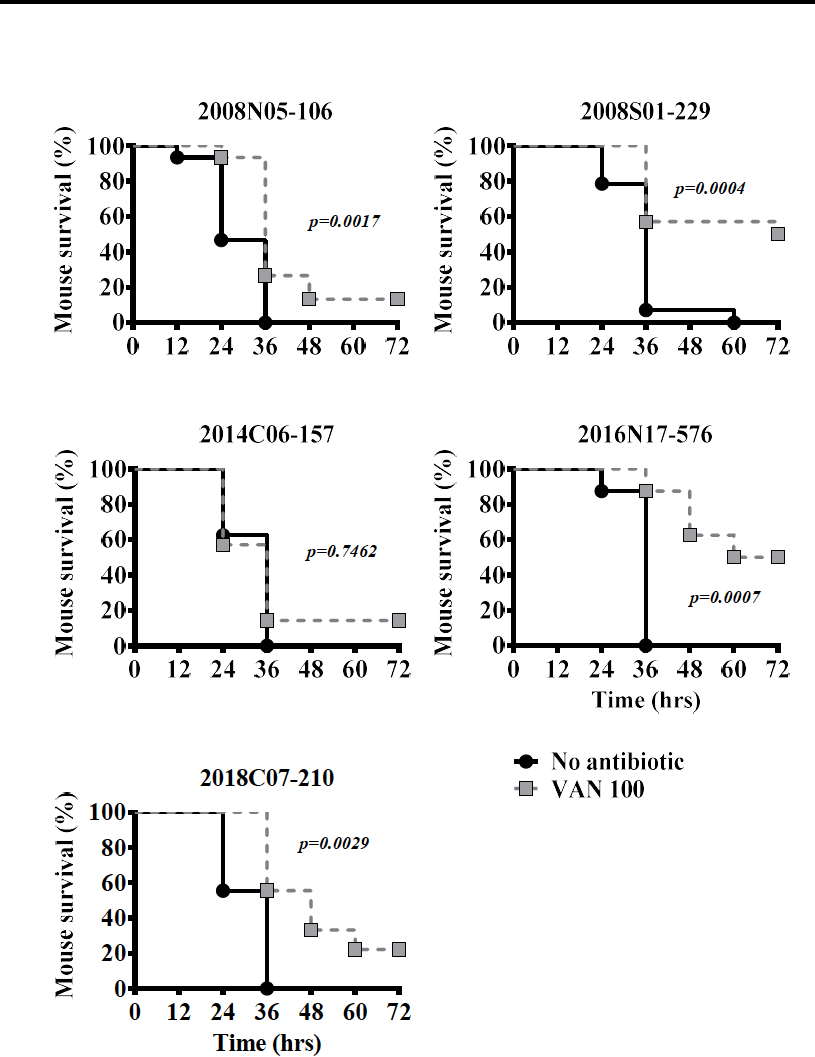
**

**Fig. S7 Bacterial loads of *Elizabethkingia anophelis* in mice given high-dose vancomycin (100 mg/kg, VAN100) in a thigh infection model.** Seven-week-old C57BL/6J mice were rendered neutropenic by intraperitoneal injections of cyclophosphamide. Subcutaneous vancomycin (100 mg/kg) was administered 2 h and 14 h after inoculation of *E. anophelis* into each of the two caudal thighs. The mice were sacrificed 24 h after inoculation. Each treatment group contained four mice. Each point represents the bacterial load of each mouse, and the bars indicate means and standard deviations.


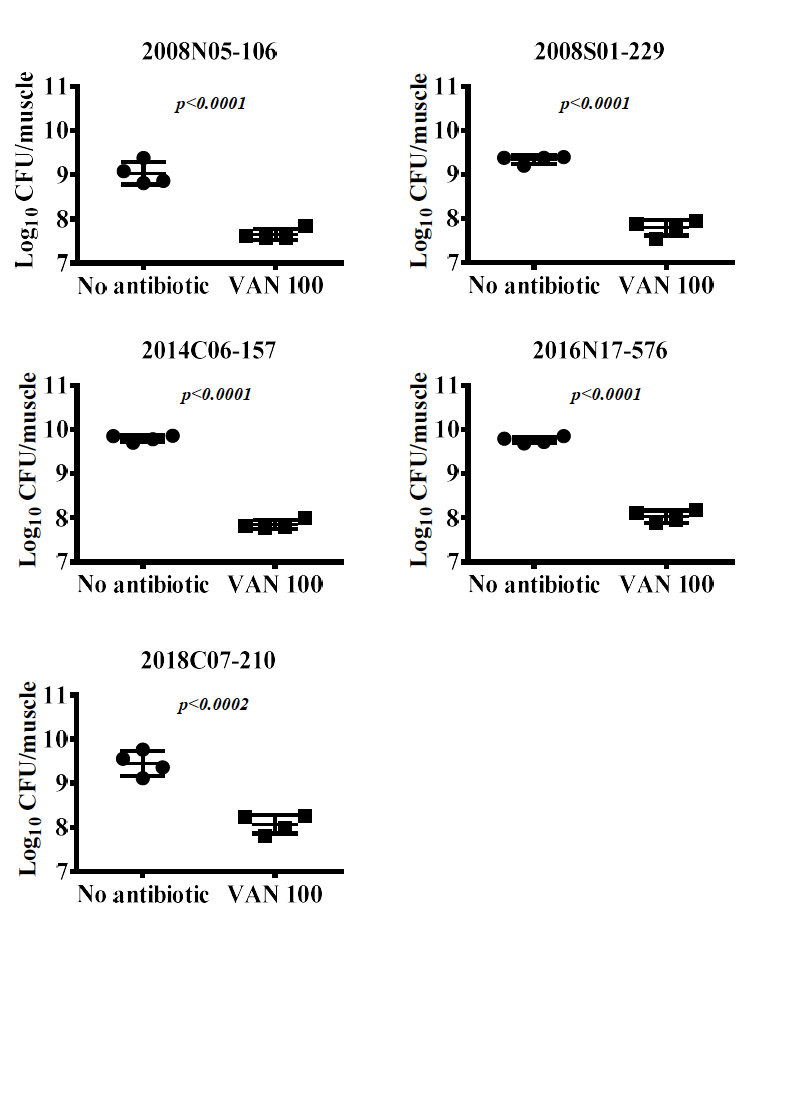


**Fig. S8 Serum drug levels in mice receiving a single dose of vancomycin or teicoplanin.** VAN25, VAN100, and TEC50 indicate doses of 25 mg/kg and 100 mg/kg of subcutaneous vancomycin and 50 mg/kg of intraperitoneal teicoplanin, respectively. Each group contained 6 to 8 mice.


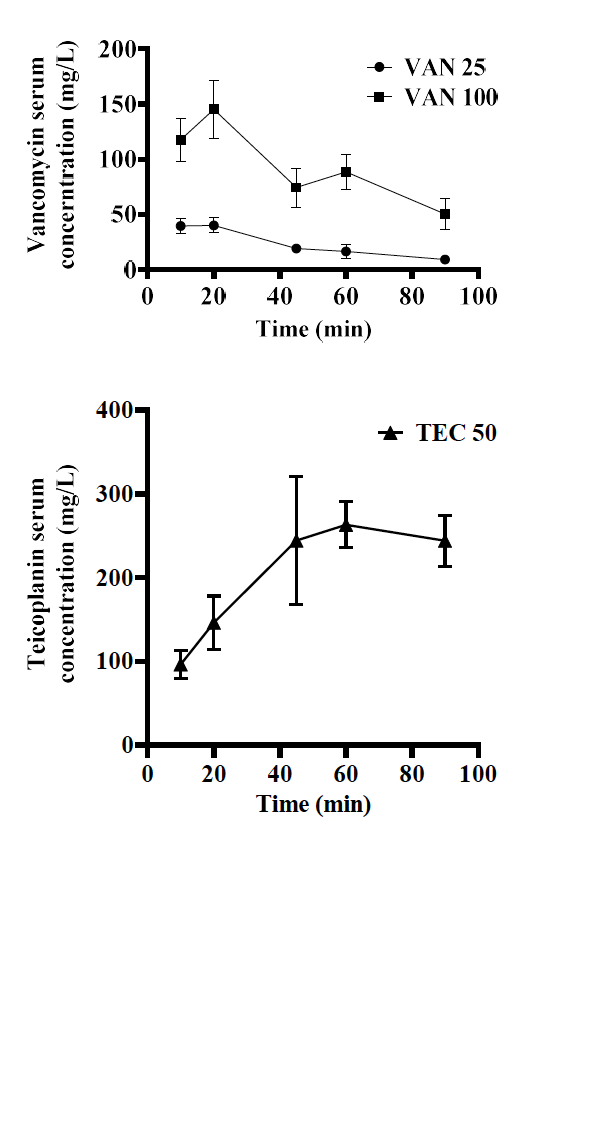


**Fig. S9 Lack of efficacy of teicoplanin (TEC) against *Elizabethkingia anophelis* in murine pneumonia and thigh infection models.** TEC was given at 50 mg/kg intraperitoneally twice daily.

**
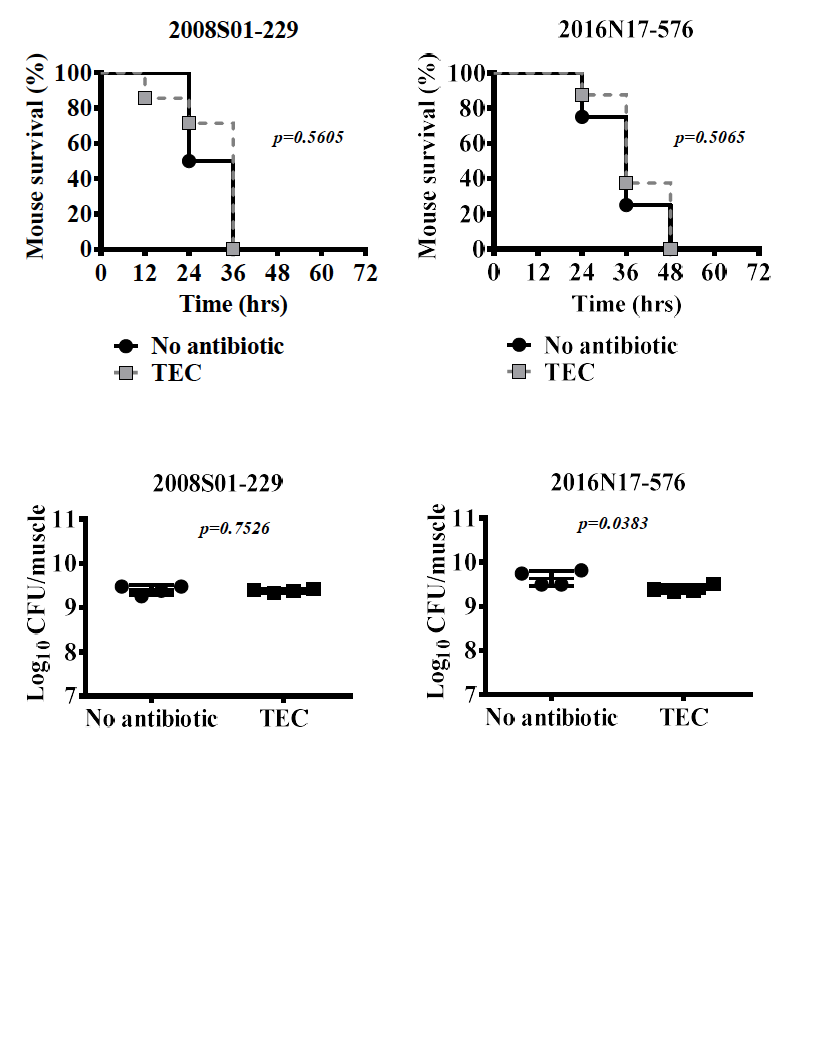
**

**Fig. S10 Lack of vancomycin (VAN) activity against *Acinetobacter baumannii* in a murine pneumonia model.** Seven-week-old C57BL/6J mice underwent intratracheal inoculation with *A*. *baumannii* (LAC-4). Two hours later, the mice were injected subcutaneously with PBS or vancomycin (25 or 100 mg/kg) twice daily. Each treatment group contained 8 mice.


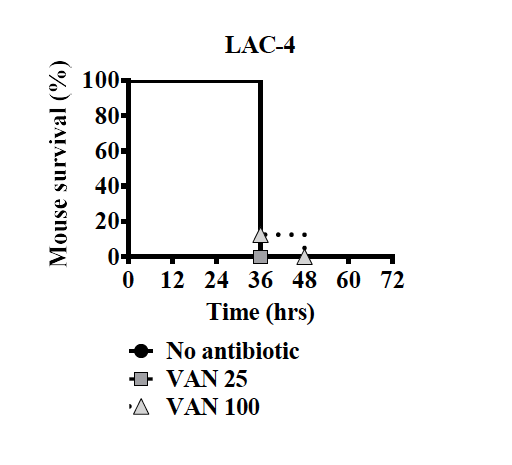

Supplement: Supplemental material — Tables S1 and S2; Fig. S1 to S10. [file spectrum.02371-25-s0001.docx]
